# Association between treatment failure in patients with early syphilis and penicillin resistance-related gene mutations of *Treponema pallidum*: Protocol for a multicentre nested case–control study

**DOI:** 10.3389/fmed.2023.1131921

**Published:** 2023-04-04

**Authors:** Hong-Fei Mi, Xu Shen, Xiao-Qing Chen, Xiao-Luo Zhang, Wu-Jian Ke, Yao Xiao

**Affiliations:** ^1^Zhongshan Hospital, Fudan University (Xiamen Branch), Xiamen, China; ^2^Zhongshan Hospital of Xiamen University, School of Medicine, Xiamen University, Xiamen, China; ^3^Dermatology Hospital, Southern Medical University, Guangzhou, China

**Keywords:** early syphilis, treatment failure, penicillin resistance, gene mutations, nested case–control study

## Abstract

**Background:**

The widespread occurrence of syphilis remains a global public health problem. Although penicillin has been recommended as the first-line therapy for syphilis for more than 70 years, treatment failure occurs in 10–20% of patients with early syphilis. Recent studies have reported varied single-nucleotide polymorphisms (SNPs) of *Treponema pallidum* related to penicillin resistance. The clinical relevance of these SNPs to treatment failure in patients with early syphilis is unresolved. In this work, a protocol is developed to evaluate the association between treatment failure in patients with early syphilis and penicillin resistance-related gene mutations of *T. pallidum*.

**Methods:**

A multicentre nested case–control study is designed, and patients who are diagnosed with early syphilis and treated with penicillin will be recruited for the study cohort. Before the first treatment, baseline information and biological specimens will be collected from the subjects, and serological tests for syphilis will be performed. Each participant will be followed up at 1, 3, 6, 9, and 12 months after the first treatment, and the clinical manifestations and serum non-treponemal test titres will be evaluated at each follow-up. Patients who will fail treatment are defined as cases, and those who will respond to treatment are defined as controls. Tests for SNPs related to penicillin-binding proteins and Tp47 will be performed in these cases and controls. Survival analysis is used performed to identify gene mutations of *T. pallidum* related to penicillin resistance and their combinations associated with treatment failure.

**Discussion:**

This protocol provides a practical clinical study design that illustrates the role of gene mutations of *T. pallidum* related to penicillin resistance in the treatment outcome of patients with early syphilis.

## Introduction

Syphilis, caused by *Treponema pallidum*, is a chronic systemic sexually transmitted disease that can involve various tissues and organs of the body. Globally, 7.1 million people were newly infected with *T. pallidum* in 2020 (1). Timely diagnosis and effective treatment are the keys to controlling the clinical progression of syphilis. If left untreated, up to one-third of patients with early syphilis would progress to an advanced stage of the disease, resulting in irreversible damage to the cardiovascular and central nervous systems, as well as death (2).

Penicillin is a β-lactam antibiotic that interferes with the function of transpeptidase enzymes [also known as penicillin-binding proteins (PBPs)] that cross-link the cell walls of actively growing bacteria. Since its first use to treat syphilis in 1943, penicillin has been recommended as the drug of choice for the treatment of all kinds of syphilis (3). Benzathine penicillin G (BPG) is recommended as a first-line therapy for early syphilis based on its long half-life and slow absorption (4). Although for more than 70 years, penicillin has achieved success in clinical resolution and preventing late sequelae for syphilis patients, treatment failure can occur with any regimen. The extreme *ex vivo* fragility of *T. pallidum* has contributed to the inability to culture it *in vitro*, with *in vivo* culture maintenance only being possible following intratesticular or intradermal inoculation of rabbits (5). Despite recent technological advances, direct *in vitro* culture of *T. pallidum* from patient samples remains unsuccessful (6). Hence, there is no gold standard for determining a cure; assessing treatment response is therefore challenging. Clinical and serological evaluations should be performed 6 and 12 months after treatment. In such evaluations, the serological response of patients (i.e., the non-specific antibody titre) is compared with the titre at the time of treatment. A decrease of more than fourfold is considered to indicate effective treatment, and a negative non-specific antibody titre after treatment is considered the best judgement of cure (7).

Clinical data show that 10–20% of patients with early syphilis do not achieve a fourfold decrease in non-specific antibody titres within 12 months after treatment, indicating treatment failure (7, 8). The effectiveness of syphilis treatment is closely related to the effectiveness of antibiotics. In recent years, non-synonymous single-nucleotide polymorphisms (SNPs) in the gene family encoding the penicillin regulatory proteins have been found in Chinese *T. pallidum* strains (*TPANIC_0500*, *TPANIC_0760*, and *TPANIC_0705*) and Lineage 3 strains (*TPANIC_0500*) (9). Homology modelling and Sorting Intolerant From Tolerant analysis revealed that one SNP (*TPANIC_0760* I415F) showed a negative impact on the structural flexibility and the binding constant for substrate stability, suggesting that this SNP may be linked to penicillin resistance in *T. pallidum*. Beale et al. selected three PBP genes, *pbp1* (TPANIC_0500), *pbp2* (TPANIC_0760), and *mrcA* (TPANIC_0705), and a putative β-lactamase, *Tp47* (TPANIC_0574), and used competitive mapping to individual gene sequences to screen for novel variants. They identified 13 non-synonymous SNPs, i.e., *pbp1* P564L, *pbp2* A366T, *pbp2* I415F, *pbp2* I415M, *mrcA* P44L, *mrcA* I487L, *mrcA* A506T, *mrcA* A506V, *mrcA* M625V, *mrcA* G708S, *mrcA* Q737R, *Tp47* S394R, and *Tp47* R312C (10). On this basis, our preliminary study found a variant in *tp0500* (P564I) to be exclusively present in isolates belonging to the SS14-like group (11). A genomic epidemiological analysis of *T. pallidum* lineages in Australia also found several SNPs related to PBPs and Tp47, including *Tp47* R107G, *Tp47* R428C, *Tp47* T27A, *mrcA* F25C, *mrcA* A566S, *mrcA* D281G, *mrcA* R167H, *mrcA* Q292R, *mrcA* V451A, *mrcA* T631P, *pbp1* C361R, *pbp1* P60R, *pbp1* P119L, *pbp1* Q449L, *pbp1* T98M, *pbp2* G361S, *pbp2* A483P, *pbp2* E535D, and *pbp2* A484E (12).

Although an increasing number of gene mutations related to penicillin resistance in *T. pallidum* have been reported, no clinical evidence of *T. pallidum* resistant to penicillin exists. Whether such widely prevalent SNPs mediate a penicillin-resistant phenotype and their possible clinical relevance remain unknown. In this protocol, a multicentre nested case–control study is designed and used to investigate the association between treatment failure in patients with early syphilis and penicillin resistance-related gene mutations of *T. pallidum*.

## Methods

### Study design and participants

A multicentre nested case–control study is planned at Zhongshan Hospital of Xiamen University, School of Medicine, Xiamen University, Dermatology Hospital, Southern Medical University, and Zhongshan Hospital, Fudan University (Xiamen Branch). Participants enrolment will take place between January 2023 and December 2023. Recruitment will cease prior to this if the desired sample size is met. The inclusion criteria for the study cohort are a diagnosis of early syphilis and treatment with penicillin.

The diagnostic criteria for early syphilis complies with the guidelines published by the Centers for Disease Control in the United States and Europe (7, 8). Briefly, early syphilis is defined as a reactive treponemal serological test result for syphilis [e.g., *T. pallidum* particle agglutination test (TPPA) or equivalent serological method] with/without a reactive non-treponemal serological test result [e.g., toluidine red unheated serum test (TRUST) or equivalent serological method] and a combination of the following findings: (1) presentation consistent with primary syphilis, which typically presents as a single painless ulcer or chancre at the site of infection but can sometimes present with multiple, atypical, or painful lesions; (2) secondary syphilis manifestations, including skin rash, mucocutaneous lesions, and lymphadenopathy; and (3) positive serological tests for syphilis without clinical manifestations during the first year after infection, indicating early latent syphilis.

Treatment for early syphilis complies with the guidelines set forth by the Centers for Disease Control in the United States and Europe (7, 8). The recommended regimen for treatment of early syphilis is 2.4 million units of BPG intramuscularly (IM) (one injection of 2.4 million units or 1.2 million units in each buttock) on Day 1. The second-line therapy option, if BPG is not available, is 600,000 units of procaine penicillin IM daily for 10–14 days.

Exclusion criteria for study participants include previous treatment with antibiotics for syphilis, allergy to penicillin, age under 14 years old, pregnancy, or coinfection with the human immunodeficiency virus.

### Sample size estimation

Based on a previously reported prevalence of penicillin resistance-related gene mutations of *T. pallidum* of approximately 11% (10, 11), with a 95% confidence level, 4% confidence interval width (two-sided), and *u*_0.05/2_ of 1.96, the calculated sample size is 940. Considering an approximately 10% dropout rate, we aim to collect data on 1,045 patients with early syphilis in the present study. Sample size estimation was conducted using PASS software (version 15.0, NCSS, USA).

### Baseline information collection

The demographic characteristics and factors associated with syphilis infection, including gender, age, nationality, occupation, educational level, history of marriage and reproduction, unsafe sexual behaviour, and the presence of other sexually transmitted diseases, will be investigated when the participants are enrolled in the cohort.

The biological specimens required for the study will be reserved before the first treatment of the subjects. They included (1) whole blood samples and (2) tissue samples (scrapings of the exudate from the chancre or skin mucosa injury or lymph node puncture fluid). Five millilitres of whole blood or tissue sample will be collected and placed in cryopreservation solution (sterilized 60% glycerol, inactivated rabbit serum, and sterile normal saline at a ratio of 5:3:7). At least two tubes of samples will be collected from each patient. Serological tests for syphilis will be performed at the time of enrolment. The samples for penicillin resistance-related gene mutations detection will be placed in a freezer at −80°C and stored in liquid nitrogen within 24 h, until the end of the follow-up.

### Follow-up and study grouping

Each participant will be followed up at 1, 3, 6, 9, and 12 months after the first treatment; the longest follow-up is 12 months. Clinical manifestations, serum TRUST titres and TPPA results will be evaluated at each follow-up.

At the follow-up, patients who will respond to treatment are defined as controls, i.e., both loss of clinical manifestations and a ≥4-fold (2 dilutions) decrease in TRUST titre or reversion to non-reactive status in the TRUST test at the 1st, 3rd, 6th, 9th, or 12th month follow-up. Follow-up will be terminated when the study participant respond to treatment; otherwise, it will continue until 12 months from the first treatment. Patients who will fail treatment (those with persistent clinical manifestations or a <4-fold decrease or any increase in TRUST titre until the 12th month of treatment) are defined as cases.

**FIGURE 1:**
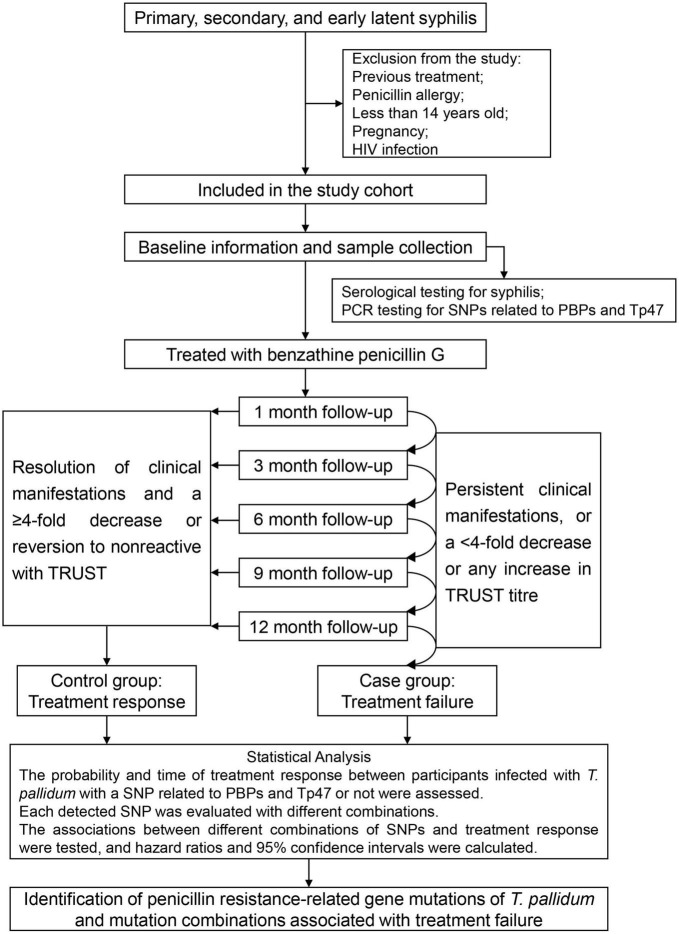
Flow chart of the multicentre nested case–control study.

A flow chart describing the multicentre nested case–control study and a list of the type of information collected at each study time point are shown in Figure 1 and Table 1, respectively.

**TABLE 1:** Information collected at each study time point.

Variables	Baseline	Follow-up (month after first treatment)				
		1st	3rd	6th	9th	12th
Demographic characteristics	√					
Unsafe sexual behaviours	√					
Other sexually transmitted diseases	√					
Serum TRUST titres	√	√*	√*	√*	√*	√*
Serum TPPA test	√	√*	√*	√*	√*	√*
PCR tests for SNPs related to PBPs and Tp47	√					
Clinical manifestations	√	√*	√*	√*	√*	√*

TRUST, toluidine red unheated serum test; TPPA, T. pallidum particle agglutination test; PCR, polymerase chain reaction; SNP, single-nucleotide polymorphism; PBP, penicillin-binding protein. *Follow-up was terminated when the study participant responded to treatment, i.e., when the patient exhibited both loss of clinical manifestations and a ≥4-fold decrease in TRUST titre or reversion to non-reactive status in the TRUST assay.

### Laboratory tests

Serological tests for syphilis will be performed on all participants using a TRUST test (Shanghai Rongsheng Biotech Co., Ltd., Shanghai, China) and TPPA tests (Fujirebio, Tokyo, Japan) according to the manufacturer’s instructions.

The samples for penicillin resistance-related gene mutations detection will be mixed with 2 × lysis buffer (20 mM Tris–HCl (pH 8.0), 0.2 M EDTA, 1% SDS) at the same volume as the original sample. Deoxyribonucleic acid (DNA) of *T. pallidum* will be extracted using the QIAamp DNA Blood Midi Kit (Qiagen Inc., Valencia, CA, USA) according to the manufacturer’s instructions. The eluted DNA will be precipitated using Dr. GenTLE^®^ Precipitation Carrier (Takara Inc., Dalian, China) and resuspended in 100 μl of buffer AE [10 mM Tris–HCl, 0.5 mM EDTA (pH 9.0)].

Four genes (*pbp1*, *pbp2*, *mrcA*, and *Tp47*) associated with β-lactam antibiotic resistance will be amplified to detect the 33 related SNPs, i.e., *pbp1* P564L, *pbp2* A366T, *pbp2* I415F, *pbp2* I415M, *mrcA* P44L, *mrcA* I487L, *mrcA* A506T, *mrcA* A506V, *mrcA* M625V, *mrcA* G708S, *mrcA* Q737R, *Tp47* S394R, *Tp47* R312C, *pbp1* P564I, *Tp47* R107G, *Tp47* R428C, *Tp47* T27A, *mrcA* F25C, *mrcA* A566S, *mrcA* D281G, *mrcA* R167H, *mrcA* Q292R, *mrcA* V451A, *mrcA* T631P, *pbp1* C361R, *pbp1* P60R, *pbp1* P119L, *pbp1* Q449L, *pbp1* T98M, *pbp2* G361S, *pbp2* A483P, *pbp2* E535D, and *pbp2* A484E. Amplification primers located within 200 nt upstream and downstream of SNPs will be designed using Primer 5.0 software for polymerase chain reaction (PCR) amplification. The sizes of the amplified products (approximately 400 bp) will be verified by 2% agarose gel electrophoresis. The PCR products will be recovered by gel purification with use of the E.Z.N.A.^®^ Gel Extraction Kit, and Sanger sequencing will be performed (commissioned by Shanghai Sangon Technology Co., LTD.). BioEdit software will be used for sequence alignment and analysis of mutation sites.

### Statistical analysis

Data collected from each research centre will be merged and analysed. Survival analysis, also referred to as time-to-event analysis, is used to evaluate the association between treatment failure and penicillin resistance-related gene mutations of *T. pallidum*. A treatment response is set as a terminal event, and the time to treatment response is recorded as the survival time. Treatment failure is recorded as censored data, and the survival time is recorded as 12 months. Kaplan–Meier analysis and the log-rank test are used to test the probability and survival time of treatment response between participants infected with *T. pallidum* with a SNP related to PBPs and Tp47 and those who are not and to draw the respective survival curves. Each detected SNP will be taken into different combinations, and univariate Cox regression analysis is used to test the associations between different combinations of SNPs and treatment failure, taking the combination of no SNPs detected as a reference, and hazard ratios and 95% confidence intervals are calculated for each combination. A multivariate Cox regression model is used to include the combinations of SNPs as well as other univariate factors associated with treatment failure in the baseline survey and to present the independent effect of the SNPs on treatment failure by eliminating the confounding effect of other factors. All statistical analyses will be performed using SPSS 25.0 for Windows (SPSS Inc., Chicago, IL, USA). A two-sided *P*-value of <0.05 is considered to indicate statistical significance.

## Discussion

The widespread occurrence of syphilis remains a global public health problem. Although penicillin has been recommended as the first-line therapy option for syphilis for more than 70 years, treatment failure occurs in 10–20% of patients with early syphilis. There is little evidence supporting the emergence of penicillin-resistant *T. pallidum*, possibly because it takes a multistep mutational process to develop penicillin resistance; nonetheless, the absence of such evidence does not mean that such resistance will not emerge. Recent studies have reported various SNPs of *T. pallidum* that appear to be related to penicillin resistance (10–12). The clinical relevance of these SNPs to the curative effect of penicillin in patients with early syphilis deserves investigation. The current study designed and used a multicentre nested case–control protocol to prospectively observe the treatment outcomes of patients with early syphilis and to identify gene mutations and combinations of such mutations in *T. pallidum* related to penicillin resistance and their association with treatment failure.

There is an existing hypothesis regarding how *T. pallidum* develops penicillin resistance-related mutants. Approximately one-fourth of patients with early syphilis suffer from neuroinvasion caused by *T. pallidum*, and some strains may be more likely to do so (13). Nevertheless, intramuscular BPG is effective in treating most early, uncomplicated cases of syphilis, although it does not result in therapeutic levels of the antibiotic in cerebrospinal fluid. When patients with early syphilis are treated with intramuscular BPG, *T. pallidum* that invades the central nervous system of such patients are likely to encounter subtherapeutic levels of antibiotics, which serve as a selection pressure for mutants with low-level penicillin resistance (14). This resistance would not be immediately detectable and would be easy to overcome if the patients are retreated with higher doses of intravenous penicillin for clinical relapse. Prior to treatment of relapsing infection, the continued transmission of treponemes with low-level penicillin resistance to new hosts and the recurrent exposure to increasing levels of penicillin of these treponemes may eventually select mutants with a clinically relevant degree of penicillin resistance (14).

The mechanisms of penicillin resistance developed by bacteria include producing β-lactamases to degrade the penicillin, acquiring additional or expressing endogenous low-affinity PBPs, altering PBPs *via* point mutations or homologous recombination, decreasing outer-membrane permeability, exporting the penicillin, or a combination of the above (15). The *T. pallidum* genome analysis predicts three putative PBPs, i.e., *pbp1* (TPANIC_0500), *pbp2* (TPANIC_0760), and *mrcA* (TPANIC_0705), but no genes encoding typical β-lactamases (16).

Recently, an abundant membrane protein of *T. pallidum*, Tp47, was found to have β-lactamase activity. This protein can degrade penicillins through a hydrolytic reaction and hydrolyse the β-lactam bonds of the corresponding penicillate products. But this reaction of Tp47 in the turnover of β-lactam antibiotics differs from other known mechanisms of the four classes of β-lactamases (17). Tp47 also shows a penicillin-binding response in which the active site of the enzyme is covalently altered (17). However, the X-ray structure of Tp47 is completely different from that of other β-lactamases or comparable penicillin-binding proteins (18). The two reactions occur at two distinct active sites, and the protein’s β-lactamase activity is more than 2,000-fold faster than its penicillin-binding reaction. The level of β-lactamase activity is high, with only strong product inhibition holding it back. Tp47 is susceptible to product inhibition at low micromolar product levels (17). As a result, if natural selection produces a variation of Tp47 that is not sensitive to product inhibition of its β-lactamase activity, *T. pallidum* will develop a novel, true penicillin resistance.

This was the first protocol to describe a clinical study to evaluate the association between treatment failure in patients with early syphilis and penicillin resistance-related gene mutations of *T. pallidum*. The multicentre nested case–control study design used in this protocol provided strong power for verification of the causal hypothesis. However, some limitations of the study should be addressed. First, DNA of *T. pallidum* that is extracted from human specimens may be mixed with a large amount of the host’s genomic DNA, and this could cause inhibition and result in low efficiency of PCR tests. Second, some patients may not have returned regularly at the time points we set after the first treatment and may have even dropped out when the clinical manifestations of their disease disappeared, resulting in loss to follow-up bias. Third, the treatment response is recorded at the 1st, 3rd, 6th, 9th, and 12th month follow-ups after the first treatment, whereas response to treatment may actually occur earlier than this. Thus, the actual time required for the treatment response to occur may be shorter than is suggested by the results of our study. Finally, treatment failure in early syphilis may be a result of multiple factors. Due to a lack of *in vitro* phenotypic validation of *T. pallidum*, it is unclear whether infection with a *T. pallidum* with resistance-related gene mutation is the immediate cause of treatment failure. In the future, it is imperative to construct *in vitro* models to verify the association between these mutations and penicillin resistance.

The protocol used in this study provides a practical measure for illustrating the role of gene mutations of *T. pallidum* related to penicillin resistance in the treatment outcomes of patients with early syphilis. A better understanding of the real causes of treatment failure contributes to devising proper treatment regimens for patients with early syphilis in clinical practice and to the prevention and control of syphilis.

## Ethics statement

This study was approved by the Institutional Ethics Committees of Zhongshan Hospital of Xiamen University, School of Medicine, Xiamen University, Dermatology Hospital, Southern Medical University, and Zhongshan Hospital, Fudan University (Xiamen Branch), and was in compliance with national legislation and the Declaration of Helsinki guidelines. Written informed consent was obtained from the patient or, from next of kin or an independent patient advocate if the patient lacked this capacity.

## Author contributions

YX and W-JK conceived and designed the study, and reviewed and approved the final protocol. H-FM and X-LZ drafted the protocol. XS and X-QC contributed to improvement of the protocol. All authors contributed to the article and approved the submitted version.
